# The role of the p90 ribosomal S6 kinase family in prostate cancer progression and therapy resistance

**DOI:** 10.1038/s41388-021-01810-9

**Published:** 2021-05-10

**Authors:** Ryan Cronin, Greg N. Brooke, Filippo Prischi

**Affiliations:** School of Life Sciences, University of Essex, Colchester, UK

**Keywords:** Prostate cancer, Nuclear receptors, Phosphorylation

## Abstract

Prostate cancer (PCa) is the second most commonly occurring cancer in men, with over a million new cases every year worldwide. Tumor growth and disease progression is mainly dependent on the Androgen Receptor (AR), a ligand dependent transcription factor. Standard PCa therapeutic treatments include androgen-deprivation therapy and AR signaling inhibitors. Despite being successful in controlling the disease in the majority of men, the high frequency of disease progression to aggressive and therapy resistant stages (termed castrate resistant prostate cancer) has led to the search for new therapeutic targets. The p90 ribosomal S6 kinase (RSK1-4) family is a group of highly conserved Ser/Thr kinases that holds promise as a novel target. RSKs are effector kinases that lay downstream of the Ras/Raf/MEK/ERK signaling pathway, and aberrant activation or expression of RSKs has been reported in several malignancies, including PCa. Despite their structural similarities, RSK isoforms have been shown to perform nonredundant functions and target a wide range of substrates involved in regulation of transcription and translation. In this article we review the roles of the RSKs in proliferation and motility, cell cycle control and therapy resistance in PCa, highlighting the possible interplay between RSKs and AR in mediating disease progression. In addition, we summarize the current advances in RSK inhibitor development and discuss their potential clinical benefits.

## Background

Prostate Cancer (PCa) is a major health problem and it is a leading cause of cancer-related death in men. In 2020, ~1.4 million new cases of PCa were diagnosed globally and nearly 500 thousand men died from the disease [1]. Treatments for organ-confined PCa include surgery and brachytherapy, and on average 87% of patients survive for 5 years or longer [2]. Tumor growth is usually dependent upon androgen, the male sex hormone, and hence patients with disease that has spread from the prostate capsule, are often treated with antiandrogens or luteinizing hormone-releasing hormone (LHRH) agonists. These aim to target the androgen signaling pathway, by blocking the Androgen Receptor (AR) or inhibiting androgen synthesis respectively. Although initially successful in the majority of patients, these therapies invariably fail and the tumor progresses to the aggressive Castration-Resistant Prostate Cancer (CRPC) stage. Few treatment options exist for CRPC and hence there is a need to identify novel therapeutic targets for this stage of the disease. A deep understanding of the signaling mechanisms that take place throughout disease progression is of paramount importance for the identification of specific and novel drug targets. One family of interest is the p90 ribosomal S6 kinases (RSKs), which lie downstream of the Mitogen-Activated Protein Kinase (MAPK) signaling pathway. These kinases have been found to be overexpressed/hyperactivated in a number of tumor types, including PCa [3]. This review summarizes our current understanding of the role of RSKs in PCa proliferation, cell cycle, cell motility, and therapy resistance.

## Origin of prostate cancer

The development of PCa is a multistep process induced by genetic and epigenetic alterations, but it is still a matter of debate as to what the exact mechanisms of PCa initiation are. The accumulation of these mutations facilitates the cells to acquire an oncogenic phenotype. A background of inherited genetic mutations can also increase the risk of developing PCa and determine its aggressiveness [4]. Similarly, several additional factors have also been associated with an increased risk of PCa development: (i) age, 75% of PCa patients in Europe are diagnosed over the age of 65 [5]; (ii) ethnicity, black men with African ancestry have a higher incidence than other ethnicities [6]; (iii) external factors such as smoking, obesity, and drug use have also been associated with PCa development [7]. These inherited genetic or accumulated epigenetic alterations can cause the development of prostatic intraepithelial neoplasia (PIN), which can progress to adenocarcinoma [8]. The types of mutations associated with PIN are predominantly chromosomal deletions, which results in loss of function of tumor suppressor genes, resulting in an increase in cellular proliferation. Further accumulation of genetic alterations drives disease progression. This is often induced by gene duplications or gene fusions, resulting in gain of function and overactivation of the existing proliferative signals [9]. As the disease progress to more aggressive stages, the number of genetic alterations increases. Indeed, analysis of PCa patient samples shows that several distinct mutations are prominent in late stage PCa, while these are rare in early stages of the disease [10]. Importantly, a number of alterations in PCa have been shown to directly or indirectly affect AR function [11]. Early disease mutations are often thought to cause dysregulation of proliferative pathways, such as MAPK or Phosphoinositide 3-Kinase (PI3K), which in turn activates the AR [12].

## The androgen receptor

**Fig. 1:**
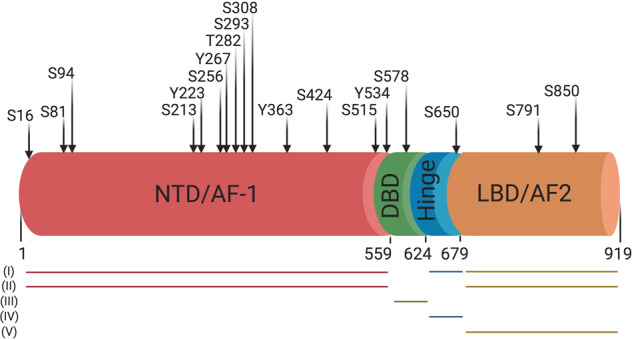
Schematic representation of the Androgen Receptor domain structure. The AR consists of an N-terminal Domain/Activation Function-1 (NTD/AF-1), DNA binding Domain (DBD), Hinge region and Ligand binding domain/Activation Function-2 (LBD/AF-2). Phosphorylation sites are highlighted with black arrows with corresponding residue numbers. Horizontal lines depict AR domains involved in (i) dimerization, (ii) transactivation and binding of co-regulators, (iii) DNA binding, (iv) nuclear localization and (v) ligand binding.

The AR belongs to the Steroid Receptor subfamily, along with the glucocorticoid (GR), mineralocorticoid (MR), oestrogen (ER) and progesterone (PR) receptors. The AR functions as a transcription factor, which recognizes and binds to specific DNA sequences, called Androgen Responsive Elements (AREs), present in the regulatory regions of target genes. The primary function of the AR is to regulate gene expression in the male sex organs through the entirety of life, which is also vital for the maintenance of the prostate gland [13]. The AR has a modular domain structure, consisting of the N-Terminal Domain (NTD), a central DNA Binding Domain (DBD) and the C-terminal Ligand Binding Domain (LBD) (Fig. 1). The DBD and LBD are connected by a linker region termed the hinge, which is important in dimerization and nuclear localization [14]. The DBD is essential for interacting with DNA, whilst the LBD, as the name suggests, binds ligands [15]. There are also additional regulatory regions in the AR, termed Activation Function 1 and 2 (AF-1, AF-2). AF-1 is located in the flexible NTD and recruits co-regulators for transcription, whilst AF-2 is located in the LBD and has been shown to regulate AF-1 activity by recruiting co-regulators which facilitate full transcriptional activity [16] (Fig. 1).

**Fig. 2:**
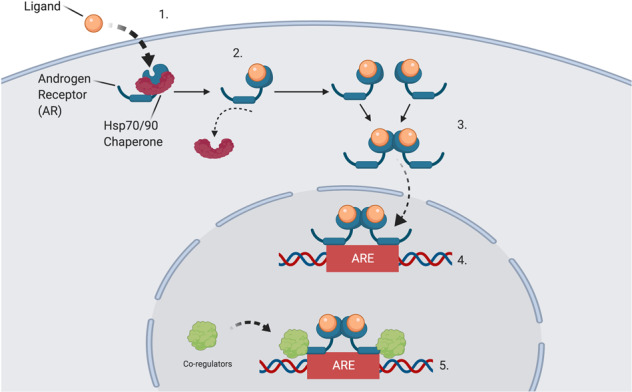
The Androgen Receptor signalling pathway. Schematic representation of Androgen Receptor activation. (1) Ligand diffuses across bilayer and binds to the AR. (2) Ligand binding causes a conformational change and dissociation of the heat shock protein (hsp) complex. (3) Activated AR monomers homodimerize and translocate to the nucleus. (4) AR dimer binds to Androgen Response Elements (AREs) located in the regulatory regions of target genes. (5) Co-regulators bind to receptor and promote target gene transcription.

Multiple studies have focused on the characterization of the AR and have shown that several steps are required for its activation (Fig. 2). In the absence of ligand, the AR is inactive and bound to chaperones (e.g., the heat shock proteins Hsp70 and Hsp90) in the cytoplasm which stabilize the AR and prevents its degradation [17, 18]. AR activation is triggered by hormone signaling. Testosterone passively diffuses through the cell membrane into the cytoplasm where it is converted into dihydrotestosterone (DHT) by 5-α-reductase. DHT binds to the AR causing a conformational change, promoting interaction between the NTD and LBD. This stabilizes the AR, the heat shock proteins dissociate, which promotes the formation of homodimers [14]. Cryo-Electron Microscopy analysis of the AR has demonstrated that surfaces of the LBD, DBD, and NTD are involved in this dimerization [16] (Fig. 1). The AR-ligand complex translocates into the nucleus, where it binds AREs [19]. To initiate gene expression, the AR recruits co-regulators and the basal transcriptional machinery [20] (Fig. 2).

The AR does not function exclusively in the male reproductive system. In fact, the AR is also known to regulate multiple secondary sites. Early work on mouse AR Knock Out models highlighted that the AR regulates bone and muscle growth, metabolism homeostasis, the cardiovascular and the hemopoietic systems, and has a neuroprotective role in the brain [21]. Furthermore, in vivo imaging of AR activity (using an AR responsive luciferase reporter system) in transgenic mice highlighted AR activity in e.g., the salivary glands, the eye (and associated glands), adipose tissue, spleen, and regions of the brain [22].

AR activity can be modulated via several different posttranslational modifications: acetylation, methylation, SUMOylation, ubiquitination and most significantly, phosphorylation [23]. Specifically, 18 phosphorylation sites have been identified on the AR, with the majority located on the AF1 (Fig. 1) [24]. Several kinases target the AR and influence its transcriptional program [25]. Among these kinases, the RSK family is of particular interest. This protein family has a wide and diverse range of cellular roles, including regulation of cell division, survival, and migration. Importantly, RSKs have been linked to cancer for their ability to mediate tumorigenesis and metastasis [26, 27]. For example, RSK2 has been shown to directly bind [28] and phosphorylate the Estrogen Receptor-α (ERα) at Ser167 [29], resulting in an increased proliferation of Breast Cancer (BCa) cells [28, 30]. Despite these initial results, very few studies have investigated the role(s) of the different RSK isoforms in hormone-driven cancers, such as PCa.

## RSK structure and activation

**Fig. 3:**
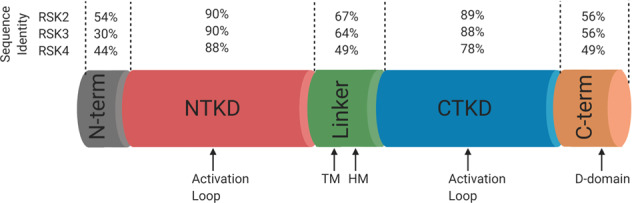
Schematic representation of the RSKs domain structure. Percentage sequence similarity of the RSK1 and RSK2/3/4 domains: N-terminal region (N-term), N-terminal kinase domain (NTKD); linker region (Linker); C-terminal kinase domain (CTKD) and the C-terminus (C-term). Motifs are highlighted in the structure with black arrows: Activation loop(s); Hydrophobic Motif (HM); Turn Motif (TM); ERK Docking Domain (D-domain).

The RSK family of serine/threonine kinases comprises four closely related proteins (RSK1-4), which are the most downstream and critical effectors of the MAPK pathway [31]. RSKs contain two distinct kinase domains in the same polypeptide chain. This originated as a result of a gene fusion event and not gene duplication since the N-Terminal Kinase Domain (NTKD) belongs to the AGC kinase group, while the C-Terminal Kinase Domain (CTKD) belongs to the CAMKII group [32]. These catalytic domains have a high sequence similarity in the four isoforms, whilst the N- and C-termini are not conserved (Fig. 3). The NTKD and CTKD are connected by a linker region of ~100 amino acids, which contains the Turn Motif (TM) and the Hydrophobic Motif (HM), two essential AGC kinase regulatory motifs. The C-terminus contains two further functional elements: the ERK D-domain (D-d) and the autoinhibitory domain, which prevents substrate and ATP binding to the CTKD [26] (Fig. 3).

RSK activation is tightly regulated and 4–5 stepwise phosphorylation events are required to reach full kinase activity [31]. The cascade is initiated by activation of Extracellular signal-Regulated Kinase 1/2 (ERK1/2), docked onto the D-d, which phosphorylates the CTKD and two residues (only one in RSK4 [33]) on the TM. The CTKD becomes active and phosphorylates the HM. Interestingly, the only known substrate of the CTKD is its own linker region. The phosphorylated HM and TM recruit and stabilize the interaction with PDK1 [34]. PDK1 then phosphorylates and activates the NTKD of RSK1-3, but not RSK4 which has autophosphorylation activity [33]. The RSKs can then phosphorylate a wide range of substrates [31, 35]. In an early attempt to identify substrate specificity, Philip Cohen’s group, using a library of peptides related to the N-terminus of glycogen synthase, showed that RSKs preferentially phosphorylate S residues over T and target substrates containing a consensus motif RXRXXpS or RRXpS [36, 37]. More recently, Roux et al. suggested that RSKs preferentially phosphorylate substrates containing the RXRXXpS sequence, highlighting the requirement for R residues at position −5 and −3 [38]. Analysis of consensus sequences recognized by individual protein kinases reveals high level of similarities among basophilic kinases, which includes the majority of known Ser/Thr protein kinases [39]. This is in line with earlier evidence showing that some AGC kinases can redundantly phosphorylate overlapping sites on substrates, further complicating the assignment of a specific substrate to a kinase within a signaling pathway [40, 41]. Indeed, several RSKs substrates can also be targeted by p70 ribosomal S6 kinase (S6K), Protein Kinase B (Akt), Protein Kinase A (PKA) and serum- and glucocorticoid-induced protein kinase (SGK) [40, 41]. While RSKs, and kinases in general, preferentially phosphorylate residues within the consensus motif, recent structural and biochemical studies have shown that substrate recognition can be driven by docking interactions with regions distant from the phosphorylation and the kinase catalytic sites. It has also been shown that temporally and spatially regulated complex interactions are required for substrate phosphorylation in vivo [42]. This is in line with evidence suggesting that RSK-isoform specific features [27, 43] and substrate recognition [44] may be driven by the non-conserved N- and/or C-termini, which are far from the kinase catalytic pocket.

An increasing number of studies have shown that RSKs play an important role in cancer progression and chemoresistance [45]. However, the lack of comprehensive studies investigating the different RSK family members has limited our understanding of the roles of the four RSK isoforms in oncogenesis [27]. This is particularly relevant in PCa; despite several studies investigating the role of RSK1/2 in the disease (discussed here below), no study has investigated the roles of RSK3/4 in PCa. This is quite surprising, since in similar hormone-sensitive cancers such as BCa, RSK4 downregulation was shown to correlate with Estrogen Receptor (ERα) upregulation and cancer progression [46, 47]. Conversely, overexpression of RSK4 was shown to inhibit doxorubicin chemoresistance, proliferation and migration, and to increase apoptosis in BCa cells [48]. Future studies should therefore aim to dissect the roles of all RSK isoforms in the same cancer type.

## The role of RSKs in prostate cancer proliferation

**Fig. 4:**
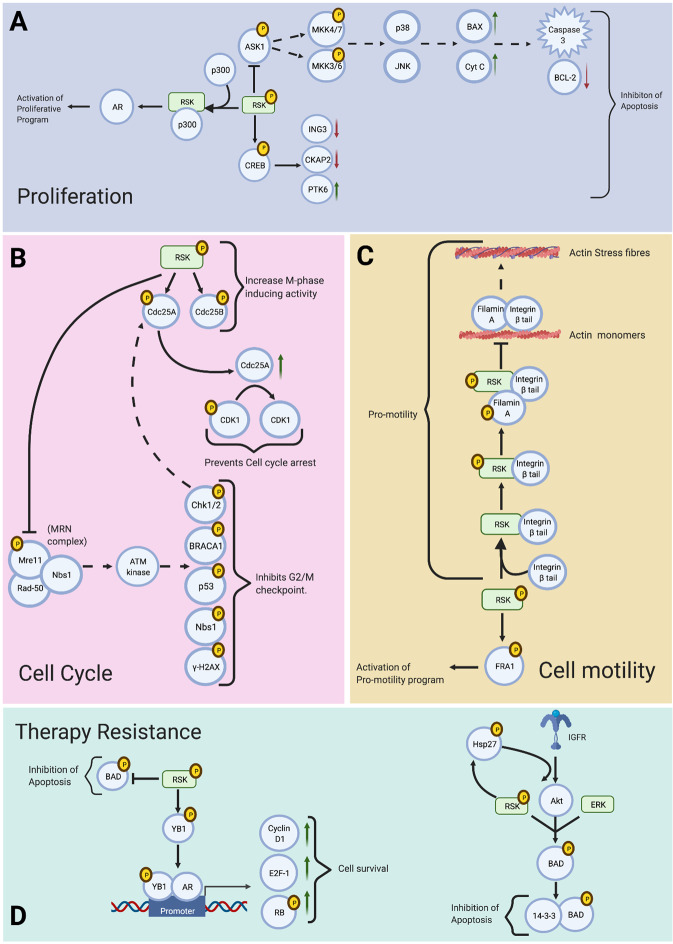
Summary of the role of the RSK family in PCa progression and therapy resistance. Schematic representation of how RSK signalling regulates PCa (**A**) proliferation, (**B**) cell cycle, (**C**) cell motility and (**D**) therapy resistance. Solid arrows depict signalling events while dashed arrows depict signalling events inhibited by RSKs. Red arrows depict a decrease in expression while green arrows depict an increase in expression. Brackets are used to summarize the overall cellular outcome.

In one of the first studies to focus on the roles of RSKs in PCa, Clark et al. [49] showed that RSK2 regulates the proliferation of the PCa cell lines LNCaP and PC3. Analysis of patient samples demonstrated that RSK1/2 protein levels were elevated 2.5–3.5-fold in PCa biopsies compared to normal prostate tissue [49]. Furthermore, overexpression and/or hyperactivation of RSK2 was linked with an increase in Prostate Specific Antigen (PSA) expression in the androgen-sensitive human prostate adenocarcinoma cell line LNCaP [49]. Elevated PSA levels correlates with PCa progression and the expression of this protein has been used as a biomarker in diagnosis and treatment decision making for decades [50]. Expression of PSA is regulated by the AR [51] and RSK2 indirectly upregulates the expression of this protease via regulation of the AR transcriptional program [49]. Earlier studies from the same group showed that RSK2 phosphorylates ERα on S167 enhancing its transcriptional activity [29]. Based on this, Clark et al. [49] investigated if RSK2 regulates AR in a similar manner. Using kinase phosphorylation assays they showed that RSK2 does not phosphorylate the two more likely phosphorylation sites of the AR, S208 and S778, indicating that the mechanisms of regulation of AR and ERα is different. Specifically, compared to the control, RSK2 only caused a twofold increase in phosphorylation of the AR, versus a 150-fold increase in phosphorylation of ERα [49]. Instead, it appears that RSK2 enhancement of AR activity is via regulation of the transcriptional co-regulator p300/cAMP Response Element-Binding protein (CREB) Binding Protein (CBP). In fact, earlier studies showed that RSKs recognize and bind the CH3 domain of p300 and antagonistically regulate transcription of CREB [52]. In a similar mechanism, but converse manner, RSK2 binding to p300 was shown to agonistically regulate the AR transcriptional program, increasing PSA expression fivefold (Fig. 4A). This was further validated using E1A, a viral oncoprotein which is known to bind p300/CBP and compete with RSK2 for the binding to p300/CBP [53].

Co-transfection of LNCaP cells with E1A, RSK2 and p300, reduced RSK2-induced transcriptional activity two- to threefold [49]. While the authors excluded direct AR phosphorylation, kinase activity experiments were carried out using short AR and ERα peptides and not the full-length proteins, which are challenging to purify [54]. Furthermore, the design of the AR peptides, used to investigate these phosphorylation events, was based only on the RSKs recognition motif RXRXXS/T. This has several limitations since RSKs have been shown to preferentially, but not exclusively, phosphorylate this motif. For example, Akt Substrate of 160 kDa (AS160) (T568), Activating Transcription Factor 4 (ATF4) (S245, S251), C-Fos (S362), Eukaryotic translation Initiation Factor 2a (eIF2A) (S52), Y-Box Binding protein 1 (YB1) (S102), p65 (S536) are phosphorylated by RSKs but lack the RXRXXS/T recognition sequence [26]. Furthermore, recent studies have shown that ERα directly binds RSK2 and that it is the shuttling of the ERα-RSK2 complex to the nucleus, and not the phosphorylation of S167 on ERα, that activates a pro-neoplastic transcriptional program [28]. It is therefore still a matter of debate if RSKs bind to the AR as a co-regulator, or bind and phosphorylate the AR.

Initial analysis of PCa patient samples showed that RSKs were highly phosphorylated and localized in the nucleus of bone metastases, which was not observed in other stages of the disease [55]. Further analysis showed that overexpression of constitutively activated RSK1 in the PCa cell line C4-2B4, injected into femurs of mouse models, enhanced tumor growth, likely by increasing PCa cell survival in the bone microenvironment. Conversely, knockdown of RSK1, and to a lesser extent RSK2, in PC3 cells derived from PCa bone metastasis caused a reduction of the osteolytic lesions when injected into mouse femurs [55]. Differently from previously published data by Clark et al. [49] showing that inhibition of RSKs using SL0101 significantly reduced LNCaP and PC3 cell proliferation, overexpression of RSK1 in C4-2B4 and knockdown of RSK1 in PC3 cells increased and decreased anchorage-independent growth respectively, but not proliferation. These differences may be linked to the culture conditions used and/or to the use of SL0101, which lacks specificity and inhibits in a non-uniform way all RSK isoforms [26].

Yu et al. [55] showed that RSK1 promotes cell survival and inhibits apoptosis by causing a reduction in phosphorylation of p38 MAPK and c-Jun N-terminal kinase (JNK), a reduction in expression of Inhibitor-of-Growth protein 3 (ING3) and Cytoskeleton-Associated Protein 2 (CKAP2), and an increase in expression of Protein-Tyrosine Kinase 6 (PTK6) [55] (Fig. 4A). Indeed, previous studies have shown that RSK2 inactivates Apoptosis Signal-regulating Kinase 1 (ASK1) by phosphorylating it on T1109 and T1326. This blocks ASK1 phosphorylation of Mitogen-Activated Protein Kinase Kinase 4/7 (MKK4/MKK7) and MKK3/MKK6, which in turn prevents activation of JNK and p38 MAPK, respectively, resulting in inhibition of apoptosis [56]. Studies on LNCaP cells have shown that JNK and p38 MAPK activates apoptosis by promoting increased expression of the apoptotic protein Bax, Cytochrome C, and concomitant increased activity of caspase 3 and reduced expression of the anti-apoptotic protein B-Cell Lymphoma 2 (BCL-2) [57] (Fig. 4A). The pro-metastatic function of RSK1 in PCa was also linked to the activation of an RSK-dependent transcriptional programme. Overexpression of RSK1 in C4-2B4 caused a reduction of ING3 and CKAP2 mRNA levels (65% and 80% respectively), and a significant (~40 fold) increase in PTK6 mRNA levels [55]. Interestingly, PTK6 is commonly found to be overexpressed in PCa patients [50]. This is linked to the ability of RSK1/2 to directly phosphoregulate CREB, promoting changes in expression of its transcriptional targets in metastatic cancers [55, 56] (Fig. 4A).

## RSK regulation of the cell cycle

A study by Chen et al. [58] was key in improving our understanding of the role of RSKs in cell cycle progression in PCa. Specifically, RSK1/2 were shown to inhibit Double Stranded Break (DSB) regulation at the G2/M checkpoint in PC3 cells. In heathy cells, DSBs in DNA are recognized by the Mre11-Rad50-Nbs1 (MRN) complex, which recruits the kinase ATM, which in turn phosphorylates several targets, such as p53, Checkpoint Kinase 1/2 (Chk1/2), Breast Cancer type 1 susceptibility protein (BRCA1), Nbs1, γ-H2AX [59–62] resulting in cell cycle arrest and DNA repair, or apoptosis [63]. Failure of this pathway leads to the accumulation of mutations or the loss of segments of chromosomal DNA, which can result in various diseases, including cancer [58, 64]. Chen et al. [58] showed that RSK2 phosphorylates Mre11 on S676, causing a direct disruption of Mre11 binding to dsDNA. This prevents Ataxia Telangiectasia Mutated (ATM) recruitment to the DSB site and its activation. Importantly, overactivation of RSK2 in PC3 cells caused a reduction in phosphorylation of ATM and a concomitant reduction in phosphorylation of Nbs1 and γ-H2AX, two ATM substrates. This results in G2/M progression and proliferation even in the presence of DNA damage. This has the potential to result in disease progression by facilitating replication of damaged DNA, which is likely to promote accumulation of further mutations [65] (Fig. 4B).

RSK1/2 have also been shown to regulate G2/M transition in PC3 cells by phosphorylating Cdc25 A and B on residues S293/295 and S353/T355 respectively (Fig. 4B). These phosphorylation events were shown to increase PC3 M-phase inducing activities [66]. The Cdc25 isoforms facilitate G2/M transition via formation of Cyclin Dependent Kinase (CDK) complexes. Regulation of the Cdc25 isoforms via phosphorylation has been attributed to several kinases, many of which are Cdc25 substrates and downstream targets of its substrates, thus promoting a feed-forward mechanism [67]. Since the RSKs are not substrates of the Cdc25 isoforms, Wu et al. [66] hypothesized that the RSKs could be responsible for initiating the G2/M feed-forward mechanism through Cdc25 phosphorylation. It remains to be fully elucidated if the RSKs regulation of Cdc25 can be interlinked with the interaction of RSK2 with Mre11. Inhibition of ATM [58] could prevent activation of the downstream kinases Chk1 and Chk2. Chk1 in turn would be unable to phosphorylate Cdc25A and promote its degradation [68]. This could cause accumulation of Cdc25A, which dephosphorylates inhibitory phosphorylation sites on CDKs (e.g., CDK1 Y14 and Y15) and in turn prevent cell cycle arrest [68] (Fig. 4B).

## The role of RSKs in cell motility

An increasing number of studies has connected RSKs to cell motility [27] and this has been linked to an increase in metastasis in PCa. RSK1/2 were shown to regulate a conserved pro-motility program in both healthy and tumorigenic prostate cells [69]. Specifically, Doehn et al. [69] used a series of selective inhibition experiments to show that RSKs regulate multilayering, wound healing, chemotaxis and cell invasiveness via the Fos-Related Antigen 1 (FRA1) transcriptional program in PCa (Fig. 4C). Interestingly, RSKs were shown to regulate FRA1 in different ways in different cell types: in MCF10A (breast) cells RSKs stimulated FRA1 expression, while in MDCK (kidney) cells RSKs phosphoactivated FRA1. This phosphorylation was not mapped onto FRA1, but based on the RSKs phosphorylation of c-Fos (a FRA1 homologue), it was proposed that RSKs phosphorylate FRA1 at S252 [69]. Importantly, FRA1 appears to regulate the expression of ~23% of the genes induced by RSKs signaling. In addition, the authors highlighted that this mechanism could contribute to a cell motility autocrine loop across all epithelial cells, which has previously been shown to exist in squamous cell carcinomas [70].

RSK2 has been shown to regulate a conserved pro-motility program across multiple cell types, including the PCa cell line DU145, through inactivation of integrin *β* [71]. This prevents the formation of the fibronectin matrix, resulting in inhibition of cell adhesion and promotion of migration. Gawecka et al. [71] elegantly showed that RSK2 interacts with the protein complex that binds to the integrin *β* tails. This presents several advantages, since activation of RSK2 allows for rapid phosphorylation of filamin A at S2152, which promotes its binding to the integrin *β* tails. The association of RSK2/filamin A with integrin *β* tails disrupts the association of the integrin complex with the actin cytoskeleton and in turn prevents the formation of actin stress fibers [71] (Fig. 4C). A similar pro-motility program has been reported in the induction of metastasis of squamous carcinoma and colon cancer cells [72]. Epidermal Growth Factor (EGF) was shown to activate the MAPK pathway and RSKs, causing a reduction in cell adhesions following the same model proposed by Gawecka et al. [71]. However, in this case EGF also stimulated an additional pathway, the Rho/Rho kinase pathway, which was shown to partially contribute to the phosphorylation of Filamin A. Inhibitor studies highlighted that the RSKs were the predominant Filamin A kinases, but in the squamous carcinoma and colon cancer cells RSKs and Rho kinase co-operatively phosphorylate Filamin A [72]. In a related study investigating the link between RSKs and metastasis in Glioblastoma (GBM), Shi et al. [73] showed that, downstream of EGF signaling, RSK2 phosphorylates Leukemia-Associated Rho Guanine exchange factor (LARG) on S1288, which in turn binds and activates RhoA GTPase, resulting in increased cellular migration and invasion. It would be interesting to investigate if this represents an RSK2-mediated feedback pathway that controls phosphorylation of Filamin A.

## The role of RSKs in therapy resistance

Inactivation or evasion of the apoptotic cell death program in PCa is often associated with Hormone Therapy (HT), a common treatment option for the disease, which targets the AR transcriptional program [74]. In fact, PCa is significantly dependent on androgens and the AR, and HT aims to reduce activation of the AR by blocking androgen production and/or binding. This is achieved by reducing the amount of circulating androgens using LHRH analogues/agonists and through the use of competitive antagonists, called anti-androgens, which prevent androgens from binding to the AR [75]. Despite initial response, the majority of PCa patients develop resistance to this treatment and the disease progresses to the therapy resistant stage, termed Castrate Resistant Prostate Cancer (CRPC). RSKs have been shown to play a role in some of the mechanisms that have been proposed to explain CRPC [76].

RSK1 has been shown to regulate the AR transcriptional program via YB1 in LNCaPs in response to androgen depletion [77]. Indeed, androgen depletion of LNCaP cells caused an increase in AR expression and enhanced phosphorylation of both RSK1 and YB1. Similar results were also observed when cells were treated with the anti-androgen drug enzalutamide [77]. Antiandrogens and Androgen-Deprivation Therapy (ADT) are common treatment options for PCa that aim to attenuate AR signaling by either reducing levels of circulating testosterone or preventing AR activation. It has been shown that RSK1 reduces the efficiency of ADT by upregulation of AR expression, promoting receptor activation even at low androgens levels. This drives PCa progression to CRPC and is a common mechanism by which therapy resistance develops in PCa [78]. In response to antiandrogen treatment RSK1 phosphorylates YB1 at S102, which promotes its nuclear localisation and binding to its cognate Y-box within the response element [79]. Dolfini and Mantovani [80] have challenged the notion that YB1 is a transcription factor able to directly bind DNA [81]. Extensive analysis of Chip-seq coupled with ribonuclease treatment suggests that YB1 may be promoting gene expression by posttranscriptional mechanisms through RNA binding. Independently from the exact mechanism of action of YB1, in LNCaPs and C4-2B cells, RSK1-YB1 regulation of the AR transcriptional program was shown to promote cell survival and reduce anti-androgen efficacy by enhancing expression of cyclin D1, E2F-1 and phosphorylation of Retinoblastoma protein (Rb) [77] (Fig. 4D). To overcome RSK-induced therapy resistance, Law et al. [82] used a cell permeable peptide, which competes with YB1 for RSK2, resulting in a reduction of YB1 S102 phosphorylation. Treatment with this peptide reduced YB1 translocation to the nucleus and inhibited the growth of PCa and BCa cell lines [82]. Interestingly, there are additional known and predicted phosphorylation sites on YB1, some of which have not yet been linked to a kinase in vivo [83]. Thus, it is possible that the RSKs could fine tune YB1 activity via several phosphorylation events which could also influence PCa progression and chemoresistance.

RSK1 was also shown to promote resistance to LY294002 (LY), a PI3K inhibitor [84]. Treatment of LNCaP cells with LY induced cell death, but co-treatment with EGF blocked LY-induced cell death in these cells. Further investigation showed that, downstream of EGF signaling, MEK/ERK/RSK1 inactivated Bcl2-associated Agonist of cell Death (BAD) via S75 phosphorylation, causing inhibition of apoptosis [84]. Zoubeidi et al. [85] showed that in PCa, Insulin-like Growth Factor I (IGF-I) signaling triggered activation of both the MAPK and PI3K/Akt pathways, promoting cell survival and a reduction in the efficacy of the pro-apoptotic drug cycloheximide. IGF-I activation of MAPK resulted in activation of RSKs, which in turns phosphoactivates Hsp27. Importantly, Hsp27 expression and phosphorylation levels were shown to correlate with PCa disease progression and were elevated in response to androgen ablation in both patient samples and PCa cell lines [85]. Phosphorylated Hsp27 can increase cell survival by potentiating the IGF-I induced activation of Akt, ERK and RSK, which leads to the phosphorylation of BAD on S75 and S99, stabilising BAD-14-3-3 complex and therefore reducing apoptosis [85] (Fig. 4D). Compared to previous studies [84] the authors identified an additional phosphorylation on S99. This is possibly the result of a convergence of the MAPK and PI3K pathways, which has been previously characterized in BCa and might also exist in PCa. Specifically, studies in BCa demonstrated that IGF can stimulate transactivation of the EGF receptor (EGFR) in the absence of EGF, leading to activation of the MAPK pathway and phosphorylation of BAD [86]. ZD1839, an EGFR inhibitor, was shown to potently induce apoptosis by blocking the MAPK activation, resulting in BAD dephosphorylation on S75. On the contrary, phosphorylation of BAD on S99 was not altered by ZD1839, confirming that this phosphorylation was independent of the EGFR-MAPK pathway and was indirectly targeted by PI3K. However, S99 was not sufficient to maintain survival when cells were treated with ZD1839. Similar cell survival mechanisms were also identified in melanoma cells, which also develop chemoresistance via RSK inactivation of BAD [87].

## RSK inhibitors and future directions

The growing body of evidence demonstrating the role of RSKs in cancer has prompted drug development studies aimed at identifying RSKs-specific inhibitors [45, 88]. The first three small molecules proposed as RSK inhibitors were FMK, SL0101 and BI-D1870 [89]. The inhibition mechanisms differ among these compounds: FMK is an irreversible RSK1/2/4-CTKD inhibitor, while BI-D1870 and SL0101 are RSKs-NTKD ATP-competitors [89]. FMK has two major limitations: it does not prevent CTKD-independent activations and it does not inhibit RSK3 [90, 91]. On the contrary, SL0101 and BI-D1870 are pan-RSK inhibitors but lack selectivity. In fact, SL0101 has an overall high EC_50_ in cells, indicating poor cellular activity [30], while high concentrations of BI-D1870 has been shown to inhibit several other kinases (i.e., PLK1, Aurora B, MELK, PIM3, MST2, and GSK3b), suggesting that many of the reported cellular effects of BI-D1870 are likely the result of non-specific interactions [92, 93]. Importantly, a recent study comparing BI-D1870 and SL0101 activity showed that, in an RSK-independent mechanism, BI-D1870 increases S6K1 phosphorylation, while SL0101 directly inhibits mTORC1. This highlights the importance of using different approaches to confirm the results obtained in studies using SL0101 or BI-D1870 to inhibit RSKs [92].

Drug design studies aimed to increase the selectivity and potency of BI-D1870, generated two novel compounds, LJI308 and LJH685 [94]. While these two molecules have lower EC_50_ values than the previous RSK NTKD ATP competitors [94], their poor pharmacokinetic profiles made them unsuitable for in vivo use [45, 88]. The first, to our knowledge, clinical trial testing an RSK inhibitor in cancer patients was announced in November 2019: Pheonix Molecular Designs initiated a Phase 1/1b clinical trial to evaluate the safety and tolerability of PMD-026 in patients with metastatic Bca and Triple Negative Breast Cancer (Clinical Trials ID: NCT04115306).

Ludwik and Lannigan [88] suggested that the development of inhibitors targeting the upstream MAPK pathway has meant that drug development for the RSKs has been limited. The MEK1/2 inhibitors trametinib, binimetinib and cobimetinib have been approved for treatment of melanoma and BRAF-mutant non-small cell lung cancer, and several more MEK1/2 drugs are currently in clinical trials [95, 96]. However, acquired resistance to these treatments is common and several studies have explored the option to target more nodes of the signaling cascade to resensitise tumor cells to treatments [95]. For example, it has been shown in vitro that dual inhibition of MEK and ERK can be used to overcome resistance to MEK inhibitors in several different cancer cell lines [97]. Using an RSK inhibitor as part of a therapeutic cocktail would present several advantages. In fact, RSKs regulate fewer processes than ERK, for example in kidney epithelial cells ERK regulates the expression of 1089 genes, while RSK regulates only 228 genes [69]. Furthermore, studies on BCa have shown that inhibition of RSK1/2 does not affect the proliferation of normal breast epithelial cells, which is an advantage compared to some of the MEK inhibitors [98]. Similarly, RSKs inhibition has been used to resensitise GBM cells to the standard chemotherapy drug temozolomide [99]. Specifically, RSK2 plays a key role in GBM progression and treatment of a GBM-derived cell line with a combination of temozolomide and BI-D1870 showed an additive antitumor effect. Importantly, the drugs had little effect on cell proliferation when used separately at the same low concentration, reducing the risk of toxicity in normal tissues [99]. Despite clear indications that RSKs are viable targets for cancer treatment/co-treatment, the use of RSKs inhibitors in the clinic is still limited. Indeed, increasing evidence suggests that different RSK isoforms perform tissue-specific and sometimes opposing functions in cancer. While no data is available for RSK3/4 in PCa, studies in lung and BCa have shown that RSK1 and RSK4 have diametrically opposing roles in diseases progression [27]. This suggests that the use of pan-RSK inhibitors may yield distinct toxicity effects and may not be optimal for anti-cancer treatments [100]. Consequently, selective RSK-isoform specific inhibition represents a more promising way to target RSKs in cancer. Future studies on RSK-isoform specific inhibitors used as monotherapies or as part of combination therapies, for the treatment of PCa, are therefore warranted.
